# Engineering the moss *Physcomitrium patens* to produce proteins with paucimannosidic glycans

**DOI:** 10.3389/fpls.2025.1605548

**Published:** 2025-07-11

**Authors:** Jessica Jonner, Benjamin Fode, Jonas Koch, Sören Boller, Paulina Dabrowska-Schlepp, Andreas Schaaf, Christian Sievert

**Affiliations:** Eleva GmbH, Freiburg, Germany

**Keywords:** paucimannosidic glycans, glyco-engineering, lysosomal acid α-glucosidase, *Physcomitrium patens*, hexosaminidase

## Abstract

The moss *Physcomitrium patens* is an advantageous host for the production of biopharmaceutical proteins, particularly due to the ease of glyco-engineering. However, the ability to produce proteins with paucimannosidic (MM) glycans in this species currently depends solely on the nature of the product. MM glycans offer benefits for some therapeutic proteins by facilitating their import into target cells via a presumed mannose receptor. Here, we describe the use of *Spodoptera frugiperda* enzymes expressed in moss to produce recombinant human lysosomal acid α-glucosidase with mainly MM glycans. We tested the expression of mannosidase type III and a hexosaminidase by varying the promoter strength and protein localization. The parental line produced recombinant α-glucosidase with no detectable MM glycans at all, whereas the weak expression of mannosidase type III targeted to the medial Golgi produced 4% MM glycans. The strong expression of a hexosaminidase targeted to the extracellular space increased the MM glycan content to 43.5%. Unlike previous attempts to express proteins with MM glycans in plants, neither of our introduced modifications interfered with growth or recombinant protein production. Our data confirm that the finely tuned expression and cellular localization of the glycosylation machinery can improve the efficiency of glyco-engineering. We also exploit the assembly of DNA fragments *in vivo*, which overcomes the limitations of traditional knock-in methods and facilitates the screening of different genetic elements. Our combined methods therefore represent the first straightforward approach allowing the production of recombinant proteins with abundant MM glycans.

## Introduction

The moss *Physcomitrium* (*Physcomitrella*) *patens* has unique advantages as a host for the production of biopharmaceuticals, including its amenity for glyco-engineering (Decker and Reski, 2020). The glycan profile of a therapeutic protein can influence its stability and functionality and is therefore a critical quality attribute in biopharmaceutical manufacturing (Strasser, 2023). Although glycosylation patterns tend to be more homogenous and stable in moss compared to other platforms, the specific glycan profile depends on the host strain and the product. For example, moss-derived human α-galactosidase A (Repleva AGAL, RPV-001), which has completed phase I clinical trials (Hennermann et al., 2019), features 57% paucimannosidic (MM) *N*-linked glycans (Shen et al., 2016). This facilitates the uptake of the drug by target cells, presumably via a yet unknown mannose receptor. In contrast, human lysosomal acid α-glucosidase (Repleva GAA, RPV-002) produced in the same host features mainly *N*-linked glycans terminating with *N*-acetylglucosamine (GlcNAc), giving the typical GnGn profile of most proteins expressed in moss (Hintze et al., 2020). It would be beneficial to develop engineered moss strains that produce GAA and other proteins with MM glycans to improve their uptake into target cells.

The GnGn profile generally found on moss proteins results from a stereotypical series of reactions in which the core Man_8_ structure is pared back to Man_5_ by mannosidase I (ManI), followed by the transfer of a GlcNAc residue by *N*-acetylglucosaminyltransferase I (GnT-I), the cleavage of two terminal mannose residues by ManII (yielding GnM), and a further transfer of GlcNAc by GnT-II (**Supplementary Figure S1**). Proteins such as AGAL that naturally display MM glycans in moss are presumed to have structures with a higher affinity for (and/or longer colocalization with) endogenous hexosaminidases, which cleave off terminal GlcNAc residues, potentially in addition to a lower affinity for GnT-II. In contrast, invertebrates such as the armyworm moth *Spodoptera frugiperda* are known for their dominant MM glycans (Shi and Jarvis, 2007), reflecting the presence of a unique ManIII that can cleave terminal mannose residues from Man_5_ before GnT-I has attached GlcNAc, and is thus able to create MM glycans directly (Kawar et al., 2001). GlcNAc residues, which form due to competition for the substrate by GnT-I, can be cleaved by several hexosaminidases. These include the unique *fdl* gene product, which is found only in insects and specifically cleaves α3-branch GlcNAc residues, as well as broad-spectrum hexosaminidases involved in *N*-glycan and chitin degradation, which act on both GlcNAc branches (Geisler et al., 2008).

In an effort to increase the proportion of MM glycans in moss, we exploited the expression of ManIII to trim oligomannose structures, and hexosaminidase to remove unwanted GlcNAc residues. We found that the expression level and localization of both enzymes was a key determinant of efficiency, and that the fine tuning of expression was necessary to optimize the MM glycan content.

## Materials and methods

### Plant material and cultivation

All strains used in this study were glyco-engineered descendants of *Physcomitrium patens* (Hedw.) Mitt. ecotype “Gransden 2004” expressing recombinant human GAA and were cultivated on standard moss medium. Detailed strain description and cultivation conditions can be found in **Text S1**.

### Cell line engineering

Transgenes were synthesized and transferred into our standard expression vector or assembled *in vivo*. Moss protoplasts were transformed via PEG-based method. Stably transformed moss clones were genotyped by PCR and transgene expression was quantified by real-time RT-PCR (qRT-PCR). Glycan profile was evaluated in 180-mL shake-flask cultures by high-performance liquid chromatography electrospray ionization mass spectrometry (HPLC-ESI-MS) analyses of in-gel digested GAA samples after sodium dodecylsulfate polyacrylamide electrophoresis (SDS-PAGE) separation of secreted proteins. For gel loading, GAA was quantified using an enzyme assay (Hintze et al., 2020). Additional details are included in **Text S1**.

### Protein production and glycan analysis

We used 1-L cultures in a stirred-tank bioreactor to represent production conditions as previously described (Hintze et al., 2020). Moss culture, GAA enzyme activity assays to determine clonal productivity, SDS-PAGE under reducing conditions, column purification, and the analysis of *N*-glycans by hydrophilic interaction liquid chromatography (HILIC) were carried out as previously described (Hintze et al., 2020). Purified GAA was quantified by size-exclusion high-performance liquid chromatography (SE-HPLC). Briefly, GAA was loaded onto a Yarra SEC-3000 column in a 25 mM sodium phosphate running buffer (pH 6.5) with 100 mM NaCl. For isocratic elution, we applied a flow rate of 0.75 mL/min for 30 min. For quantification, the peak area was analyzed using freely available GAA (Myozyme) as a reference.

## Results

**Figure 1:**
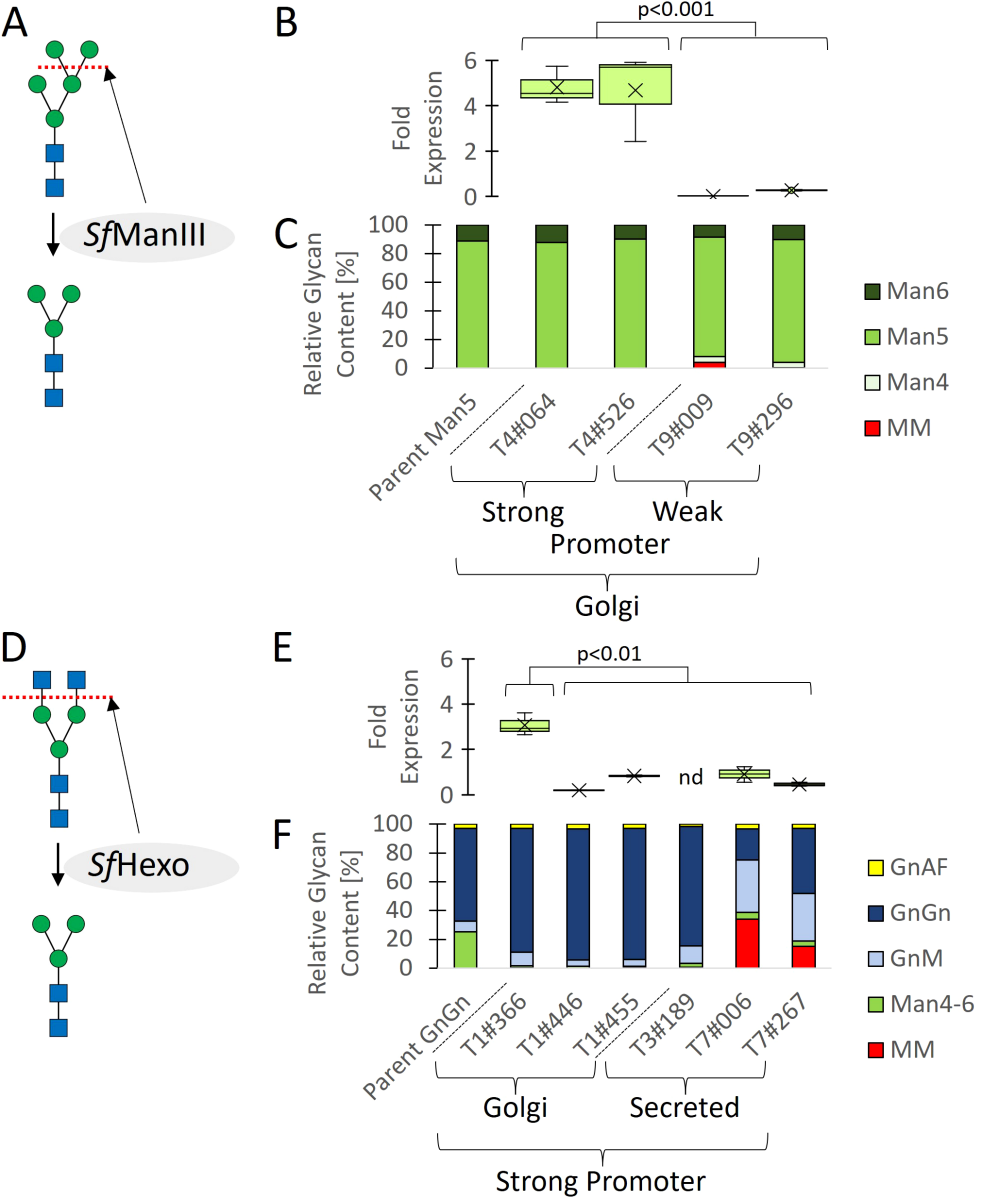
Cell line engineering, screening, and glycan profiling. Two parental lines expressing GAA with mainly GnGn (Pp_P_GAA-1#001) or Man_5_ (Pp_P_GAA-1#007) glycans were glyco-engineered to favor MM glycans by expressing *Sf*ManIII or *Sf*Hexo under the control of strong or weak promoters, and with targeting to early or late Golgi vessels using a transmembrane domain, or without a domain fusion for secretion. **(A)** *Sf*ManIII was used to cleave Man residues from Man_5_ glycans. **(B)** Transcript levels. **(C)** Proportions of different glycans. **(D)** *Sf*Hexo was used to cleave GlcNAc residues from GnGn glycans. **(E)** Transcript levels. **(F)** Proportions of different glycans. Transcript levels are shown as boxplots and are reported as 2^-ΔC^_T_ values (Schmittgen and Livak, 2008) representing the relative fold change related to endogenous *actin* mRNA (*n* = 4 including two technical and two biological replicates, nd = not detected; significance of difference between high and low transgene expression determined using a Mann–Whitney U-test; **Text S1**). Glycan proportions in **(C, F)** were determined by HPLC-ESI-MS for the quantification of glycosylated peptides.

**Figure 2:**
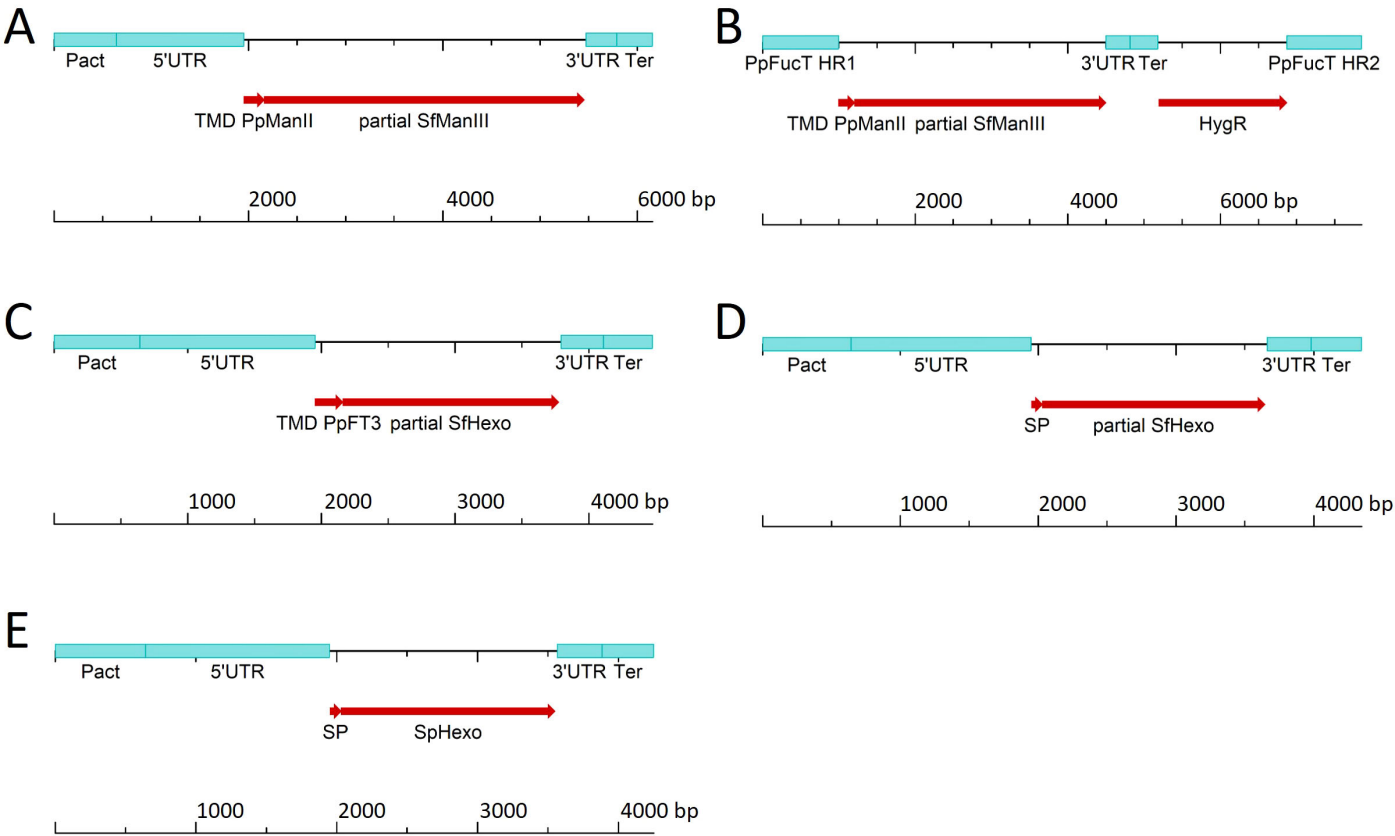
Linear DNA constructs used to express *Sf*ManIII under the control of **(A)** the strong *actin* promoter (P*act*) or **(B)** the weak fucosyltransferase promoter (*fucT*) and to target the *Sf*ManIII product to the *cis*-Golgi, to target *Sf*Hexo to **(C)** the *trans*-Golgi or **(D)** for secretion, and **(E)** to target *Sp*Hexo for secretion. All major elements are specified in **Supplementary Table S1**. HygR, hygromycin-resistance cassette; SP, signal peptide; Ter, terminator; TMD, transmembrane domain; UTR, untranslated region.

We expressed *S. frugiperda* ManIII in the high-performance GAA-producing moss line Pp_P_GAA-1#007, which has been modified to eliminate the *xylT* and *fucT* gene products needed for the synthesis of plant-specific α-1,3-fucose and β-1,2-xylose residues (Koprivova et al., 2004) as well as GnT-I, thus yielding high-mannose *N*-linked glycans mainly with the structure Man_5_ (**Figure 1A**). We expressed ManIII under the control of the strong endogenous moss *actin* promoter and fused it to the transmembrane domain of endogenous moss ManII for localization to the Golgi, where its substrate is found (**Figures 2**, **S1**). Having verified transgene integration and transcription (**Supplementary Figures S3**, **1B**), we screened for MM glycans in GAA recovered from the supernatant of shake-flask cultures following protein separation by SDS-PAGE. However, HPLC-ESI-MS analysis did not detect any MM glycans (**Figure 1C**).

Overloading the protein secretion machinery can be detrimental (Torres et al., 2022), so we expressed the ManIII under the control of the weaker *fucT* promoter by using homologous recombination to create a single-copy knock-in strain at the disrupted *fucT* locus. To provide an expression cassette for the genetic transformation of moss cells, we delivered small PCR fragments, representing the required genetic elements and coding sequences (**Supplementary Figure S2**), into the cells and used the *in vivo* assembly capabilities of moss for stable genetic transformation. With this approach, we achieved a knock-in success rate of 15.8% (**Supplementary Figure S4**). Transcript analysis confirmed that the *SfManIII* transgene was expressed at a lower level when driven by the *fucT* promoter, verifying the knock-in strategy (**Figure 1B**). HPLC-ESI-MS analysis revealed the presence of a small quantity of MM glycans in clones T9#009 (4%) and T9#296 (0.1%), relative to the sum of all identified and glycosylated GAA peptides (**Figure 1C**).

We previously generated Repleva GAA with MM glycans by using the bacterial hexosaminidase β-*N-*acetylglucosaminidase S from *Streptomyces plicatus* (*Sp*Hexo) for the modification of purified GAA *in vitro* (Hintze et al., 2020). However, when we expressed *Sp*Hexo in the high-performance GAA-producing moss line Pp_P_GAA-1#001, which has been modified to eliminate the *xylT* and *fucT* genes (Koprivova et al., 2004) but retains GnT-I and therefore synthesizes mainly GnGn glycans (**Figure 1D**), we were unable to detect *Sp*Hexo transcription in any transgenic lines after several attempts to achieve stable transgene integration (data not shown). This suggests the product is toxic (**Text S3**). The addition of a signal peptide targeting the protein for secretion to avoid interference with Golgi-resident host proteins did not resolve this issue. We therefore expressed the broad-spectrum *S. frugiperda* hexosaminidase (*Sf*Hexo) in the same parent strain, this time fusing the protein to the transmembrane domain of FucT and following the same expression strategy as described above for *Sf*ManIII. We used the strong *actin* promoter for *Sf*Hexo and obtained clones with varying expression levels during screening (**Figures 1E**, **S5**), but again detected no MM glycans by HPLC-ESI-MS (**Figure 1F**). Localization within the late Golgi may prevent enzyme processing for activation or limit the colocalization of *Sf*Hexo with its substrate, so we expressed a soluble *Sf*Hexo without a transmembrane domain instead. This finally yielded two clones with 34.1% (T7#006) and 15.2% (T7#267) MM glycans, respectively (**Figure 1F**).

**Figure 3:**
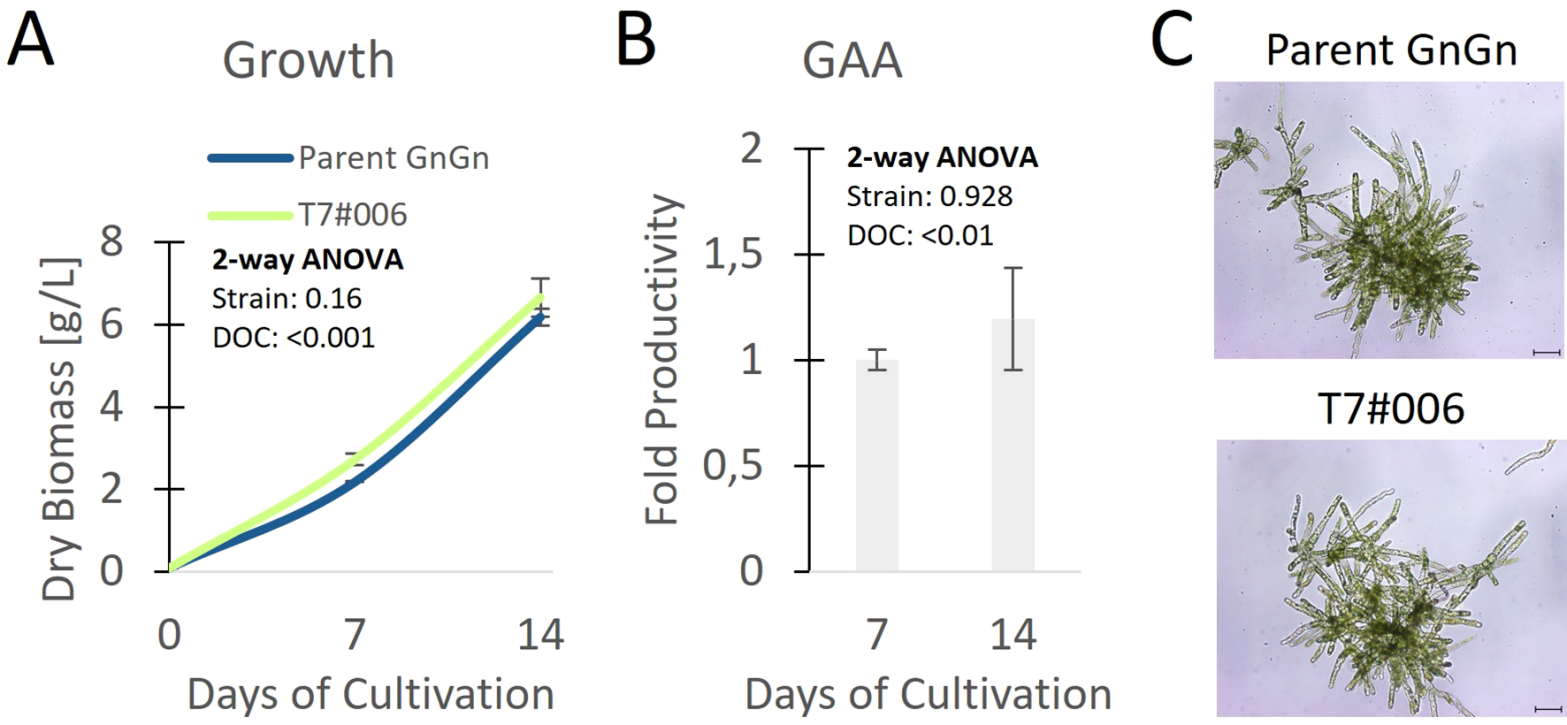
Clone with the highest proportion of MM glycans compared to the parental line showing **(A)** growth, **(B)** relative fold change of productivity related to parent, and **(C)** morphology on day 7 in a stirred-tank bioreactor (*n* = 3 technical replicates, error bars represent standard deviations). Statistical significance of differences between the parent strain and T7#006 on different days of cultivation (DOC) was determined by two-way ANOVA (**Text S1**); p values are for comparisons between strains and DOC. Scale bar represents 100 µm.

**Figure 4:**
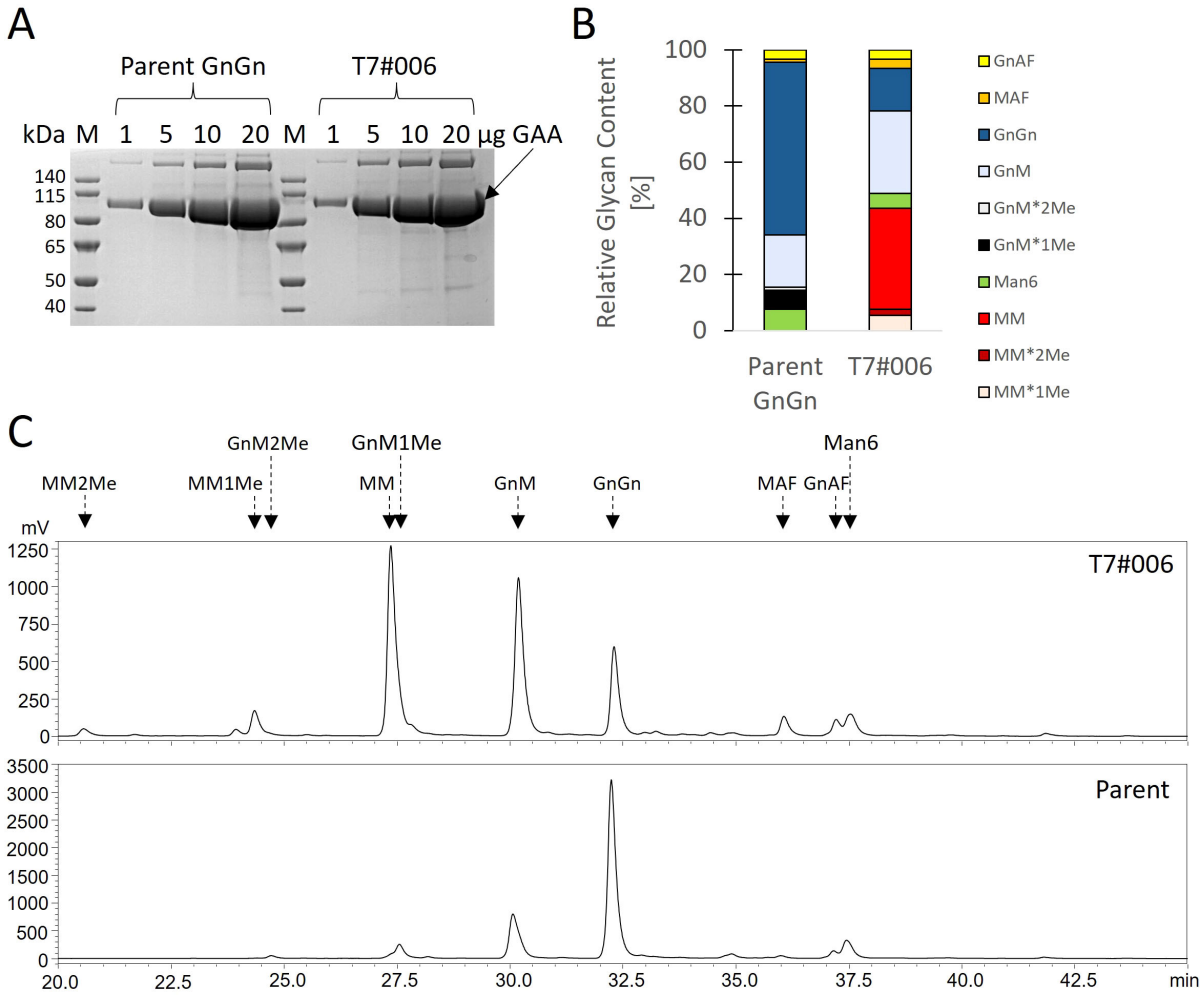
Glycan profile of GAA produced in a stirred-tank bioreactor. **(A)** SDS-PAGE showing amounts of purified GAA to estimate sample purity. **(B)** HILIC analysis and **(C)** proportions of cleaved glycans.

To quantify the portion of MM glycans in a production setting, we repeated the cultivation in a stirred-tank bioreactor using the best-performing strain (T7#006) along with the parent strain. During a 14-day cultivation experiment, both strains showed comparable morphology, growth and GAA production (**Figures 3A-C**). We extensively purified the GAA (**Figure 4A**, **Text S2**) to enable product-specific quantification of the cleaved glycans by HILIC, confirming that the GAA features up to 43.5% MM glycans (including methylated derivatives) and the GnGn content fell from 61.5% in the parental strain to 15.2% in the engineered line (**Figures 4B, C**).

## Discussion

Glycosylated therapeutic proteins usually feature complex glycans terminated with GlcNAc or sialic acid residues (Kim et al., 2009; Shin et al., 2017). However, paucimannosidic (MM) *N*-glycans facilitate the uptake of proteins via mannose receptors, as shown for AGAL (Shen et al., 2016) and potentially for other lysosomal storage disease-associated proteins such as GAA (Platt et al., 2018). Recombinant proteins with MM glycans can be produced by cleaving off GlcNAc residues *in vitro* using a bacterial hexosaminidase, but this adds a process-related impurity that must be removed in a subsequent step, increasing costs (Hintze et al., 2020). The direct formation of MM glycans in the production host would be more elegant, as achieved when using the baculovirus expression system in Sf9 insect cells due to their prominent hexosaminidase activity (Bonten et al., 2004; Shi and Jarvis, 2007). Plant expression hosts typically produce complex-type *N*-linked glycans terminated with GlcNAc residues (Hanania et al., 2017; Hintze et al., 2020; Sariyatun et al., 2021; Tschongov et al., 2024), although MM is found more rarely (Shaaltiel et al., 2007; Shen et al., 2016). Strategies that favor MM glycans include directing recombinant proteins to the vacuole, which contains hexosaminidases (Tekoah et al., 2015), or using the Arabidopsis *alg3* mutant, which inhibits glycan maturation in the Golgi, although the latter induces ER stress and reduces overall yields (Sariyatun et al., 2021). We decided to equip our moss platform with the enzymes needed to produce MM glycans to expand our glyco-engineering toolbox and benefit from our previous achievements, i.e. efficient secretion of a recombinant protein lacking plant-specific xylose and fucose residues (Hintze et al., 2020). The hereby tested *in vivo* assembly approach offers a straightforward approach to facilitate cell line engineering (King et al., 2016; **Text S3**).

*Sf*ManIII was suitable for the production of recombinant GAA with MM glycans but it was important to tune the expression levels carefully to avoid overloading the secretion machinery (Torres et al., 2022). We fused *Sf*ManIII to the transmembrane domain of moss ManII to ensure localization in early Golgi vessels (Strasser et al., 2006) but detected only traces of the product. Glycosyltransferase activity is finely tuned by enzyme localization and multimerization (El-Battari et al., 2003). Given that *Sf*ManIII is a type II α-mannosidase that forms multimers (Kawar et al., 2001; Kuokkanen et al., 2007; Nemčovičová et al., 2013), overcrowding may reduce its activity by constraining interactions involving the transmembrane and lumenal domain by changing membrane curvature, reducing the lateral diffusion rate, and compressing the distance between subunits (Löwe et al., 2020; Welch and Munro, 2019).

We achieved the highest proportion of MM glycans by expressing *Sf*Hexo under the control of a strong promoter and secreting it to the extracellular space. When we targeted the late Golgi by fusing the lumenal domain of *Sf*Hexo to the transmembrane domain of FucT (Fitchette-Lainé et al., 1994), we observed no activity at high or low expression levels. Native *Sf*Hexo is known to localize in secretory vesicles and outside the cell (Aumiller et al., 2006; Tomiya et al., 2006). We therefore cannot exclude the possibility that *Sf*Hexo exists as a pro-enzyme that must be processed as it moves through the secretory pathway to gain full activity (Tomiya et al., 2006). This is supported by our observation that only the secreted version of the enzyme was active. Another reason could be a prolonged co-localization of *Sf*Hexo with its secreted substrate GAA or a combination of both.

In conclusion, our results highlight the importance of appropriate expression levels and protein localization for components of the glycosylation machinery when optimizing the glyco-engineering of recombinant proteins (Strasser, 2023). Further engineering attempts, by testing other enzymes, different subcellular compartments and expression levels, may determine whether the MM content can be increased even more or if there is a maximum that moss can tolerate. Each attempt at glyco-engineering has the potential to alter host cell proteins and may negatively affect host physiology. However, the moss strain reported here shows no adverse changes in morphology, growth, or productivity, in agreement with many previous glyco-engineering experiments (Bohlender et al., 2020; Koprivova et al., 2004; Parsons et al., 2012; Shen et al., 2016; Tschongov et al., 2024). This confirms the amenability of moss for glyco-engineering, as also reported in other plants such as tobacco (Kittur et al., 2020).

## Data Availability

The datasets presented in this study can be found in online repositories. The names of the repository/repositories and accession number(s) can be found in the article/**Supplementary Material**.
