# Supplementary material for: Engineering the moss Physcomitrium patens to produce proteins with paucimannosidic glycans
Source: Front Plant Sci. 2025 Jul 11;16:1605548. doi: 10.3389/fpls.2025.1605548 (PMC12289595; doi:10.3389/fpls.2025.1605548)
Supplement: Supplementary file 1 [file DataSheet1.pdf]

Text S1 Materials and methods

Plant material and cultivation

Line Pp_P_GAA-1#001 produces GAA with predominantly GnGn glycans whereas line Pp_P_GAA-1#007 produces GAA with mainly high-mannose *N*-linked glycans due to the inactivation of a gene at locus Pp3c22_17330 encoding *N*-acetylglucosaminyltransferase (Hintze et al., 2020). Both of these parental lines are devoid of plant-specific fucose and xylose residues on *N*-linked glycans because the corresponding transferases encoded by genes at loci Pp3c11_22030 and Pp3c14_26580 have been knocked out (Koprivova et al., 2004). Biomass was cultivated axenically in mineral medium as previously described (Decker et al., 2015; Niederau et al., 2024). Cultures were maintained at 24 °C, with 16-h photoperiod (80 µmol m^{-2} s^{-1} photons) in flasks shaking at 120 rpm, and were subcultured every 7 days.

Cell line engineering

SpHexo, *SfHexo,* and *SfManIII* gene sequences were fused to partial 5′ untranslated regions (UTRs) from the moss *actin* gene including subcellular localization signals as needed *in silico* (Baur et al., 2005; Fitchette-Lainé et al., 1994; Strasser et al., 2006). The designed sequence fragments were synthesized and assembled by GeneArt Gene Synthesis Services (Thermo Fisher Scientific). Verified synthetic DNAs (Sanger sequencing by GeneArt) were transferred to moss expression vectors based on plasmid pRT99 (Toepfer et al., 1988; Weise et al., 2006), which contains the moss *actin* promoter, using restriction enzymes *Bst*BI and *Sac*I, in order to create a complete, functional upstream promoter cassette. DNA constructs expressing *Sf*ManIII under the control of the weak moss *fucT* promoter and targeting the protein to the medial Golgi were generated by PCR and assembled *in vivo* as described by King et al. (2016).

Protoplast isolation and genetic transformation was carried out as described by Decker et al. (2015) with minor modifications. Briefly, shake-flask cultures were maintained in mineral media. Seven days before transformation, a pre-culture for transformation was inoculated with 0.1 g/L dry weight of moss biomass. Protoplasts were isolated after digestion with 1% (w/v) Driselase for 3 h. For marker-free cell line engineering, 25 µL of adjusted protoplast suspension was mixed with 1.5 µg linear DNA and transformed without antibiotic selection. To isolate single clones, the regenerated pool of transformed clones was diluted with regeneration medium to ~40 live and dividing cells/mL before plating. Vitality and cell division was accessed by microscopy. For *in vivo* assembly, 800 µL of the protoplast suspension was mixed with 40 µg of equimolar DNA fragments and cultivated in the presence of hygromycin for one round of selection without further dilution of the culture before plating. Regenerated clones were cultivated on solid medium (omitting the commonly used cellophane sheets) for 14 days. For screening, clones were cultivated individually on solid medium in 48-well plates and genotyped by PCR (Table S2, Figures S2–S5). Positive clones were maintained in shake flasks. All subsequent tests, including PCR to verify full-length transgene integration, expression analysis by

qRT-PCR, and glycan analysis, were conducted with samples derived after several rounds of subculture in shake flasks, confirming the genetic stability of the selected clones.

Genomic DNA extraction

Harvested, part-dried moss material was flash-frozen in liquid nitrogen and stored at –75°C until preparation. Frozen material was milled in a mixer mill (MM300, Retsch). Genomic DNA was extracted from 100-mg samples dissolved in 300 µL Plant DNAzol mix (Thermo Fisher Scientific) containing 3 µl RNase A. The tubes were inverted five times to mix, then incubated for 5 min at 25 °C, shaking at 600 rpm. We added 300 µL trichloromethane, mixed and incubated as above, and then separated the phases by centrifugation (13,000 × g, 10 min, room temperature). The supernatants were mixed with five volumes of Buffer 1 from the DNeasy Plant kit (Qiagen) and all subsequent steps were carried out according to the manufacturer's instructions using pre-heated elution buffer (65 °C).

RNA extraction, cDNA synthesis, and qRT-PCR

Frozen moss material was prepared and milled as above, and total RNA was extracted from 30-mg samples using the RNeasy kit (Qiagen) including the DNase treatment step to remove genomic DNA. We then prepared cDNA using the SensiFAST kit (Meridian Bioscience) according to the manufacturer's instructions. Moss cDNA samples were analyzed by qRT-PCR by Microsynth using primers and probes based on the target gene sequences we provided (Table S2). A minimum of 95% efficiency on the synthetic template was applied as an acceptance criterion. No reverse transcriptase controls to detect residual genomic DNA and no template controls, i.e. water, to detect contamination have been run in parallel. The melting curve of the PCR product was used to evaluate specificity of the reaction.

Glycan analysis for clone screening

Moss strains were screened for paucimannosidic *N*-linked glycans by HPLC-ESI-MS using in-gel digested GAA samples. Proteins secreted into the culture medium were separated by SDS-PAGE (Hintze et al., 2020) and GAA bands were excised for further processing. Proteins were *S*-alkylated with iodoacetamide and digested in-gel with trypsin (Promega) and/or GluC and/or chymotrypsin (both Roche). The digested samples were loaded onto a BioBasic-18 C18 column (150 × 0.32 mm, 5 µm; Thermo Fisher Scientific) using 80 mM ammonium formate buffer as the aqueous solvent A and 100% acetonitrile as solvent B. We established a gradient from 5% to 32% B in 35 min, and 32% to 75% B in 15 min to elute large peptides at a flow rate of 6 µL/min. The peptides were detected by quadrupole time-of-flight (QTOF)-MS using a maXis 4G instrument (Bruker) equipped with the standard ESI source in positive-ion data-dependent acquisition (DDA) mode, switching to MS/MS mode for elution. MS scans were recorded in the range 150–2200 Da, and the four highest peaks were selected for fragmentation. Alternatively, the samples were separated by nano-flow HPLC (300 nL/min) on an Acclaim PepMap column (25 cm × 0.075 mm ID, 2 µm particle size; Thermo Fisher Scientific) followed by detection using the Bruker maXis 4G equipped with a captive spray ESI

source in positive ion/DDA mode as above. MS scans were recorded in the range 150–2200 Da, and the six highest peaks were selected for fragmentation. We used the ESI calibration mixture (Agilent) for instrument calibration. Manual glycopeptide searches were carried out using DataAnalysis 4.0 (Bruker) followed by conversion to mgf files, which are suitable for MS/MS ion searches with Global Proteome Machine (GPM). To quantify the different glycoforms, the peak areas of extracted ion chromatograms representing the first four isotopic peaks were summed using Quant Analysis (Bruker). We detected and quantified the GAA glycopeptides LENLSSSEMGYTATLTR, QVVENMTR, GVFITNETGQPLIGK, NNTIVNELVR and VTVLGVATAPQQVLSNGVPVSNFTYSPDTK.

Statistics

Gene expression data were normalized to endogenous *actin* using the $2^{-\Delta C_T}$ method (Schmittgen and Livak, 2008). The normalized data were grouped in high and low expression categories following visual inspection. The statistical significance of differences between groups was calculated using a Mann–Whitney U-test in R v4.4.2. Growth and productivity data from stirred-tank bioreactor runs were tested by two-way analysis of variance (ANOVA) for repeated measurements in R v4.4.2.

Text S2 Results

SDS-PAGE

SDS-PAGE was used to evaluate the purity of GAA samples as described by Hintze et al. (2020). The additional bands below GAA at ~110 kDa probably represent host cell proteins (HCPs) that have not been removed during purification because we selected a purification strategy that was fast and robust rather than the most efficient. The identity of HCPs has not been determined. The band at ~220 kDa is a product-related impurity (GAA dimer) as confirmed by MS (data not shown).

Text S3 Discussion

*Sp*Hexo Expression

We selected *Sp*Hexo as a candidate to introduce MM glycans in moss because this hexosaminidase cleaves GlcNAc residues efficiently *in vitro* (Hintze et al., 2020; Mark et al., 1998). However, none of the transgenic clones we recovered expressed *SpHexo* mRNA or *Sp*Hexo protein. The *actin* promoter, UTR and signal peptide function effectively for other transgenes like *SfHexo*. It is unlikely that the moss genetic elements are incompatible with bacterial genes because they allowed the expression of other bacterial genes, including antibiotic resistance genes. However, the particular combination of *SpHexo* driven by the *actin* promoter and UTR fused to a signal peptide sequence has not been tested before. We speculate that folding inside the endoplasmic reticulum (ER) is not compatible for a protein optimized by evolution to fold in the bacterial cytosol. The *Sp*Hexo sequence contains two cysteine residues and one *N*-glycan acceptor site (Gupta and Brunak, 2002) that are not modified in bacteria but may be targeted for disulfide bond formation and glycosylation, respectively, in the moss ER lumen. This would cause misfolding and severe ER stress that inhibits growth and reduces vitality (Crosti et al., 2001) and may explain why we were unable to detect any clones expressing *Sp*Hexo during screening.

Cell line engineering using *in vivo* assembly

Our *in vivo* assembly approach achieved a knock-in success rate of 15.8%, which is in line with the 14.8–22.2% reported by King et al. (2016). However, knock-in rates in moss can vary from < 0.1% to > 10% depending on the genomic context and the nature of the transformation construct. As an alternative, CRISPR/Cas9 technology is now well established in moss (Zhu, 2021). We frequently target genes using CRISPR/Cas9 with a success rate of 80–95 % (data not shown) and Collonnier et al. (2017) reported a success rate near to 100%. The combination of *in vivo* assembly and CRISPR/Cas9 would allow the targeting of any gene while screening different genetic elements delivered into the cell as fragments.

Text S4 References

Baur, A., Kaufmann, F., Rolli, H., Weise, A., Luethje, R., Berg, B., Braun, M., Baeumer, W., Kietzmann, M., Reski, R., *et al.* (2005). A fast and flexible PEG-mediated transient expression system in plants for high level expression of secreted recombinant proteins. *J. Biotech.* 119, 332–342.

Collonnier, C., Epert, A., Mara, K., Maclot, F., Guyon-Debast, A., Charlot, F., White, C., Schaefer, D.G., and Nogué, F. (2017). CRISPR-Cas9-mediated efficient directed mutagenesis and RAD51-dependent and RAD51-independent gene targeting in the moss *Physcomitrella patens*. *Plant Biotechnol. J.* 15, 122–131.

Crosti, P., Malerba, M., and Bianchetti, R. (2001). Tunicamycin and Brefeldin A induce in plant cells a programmed cell death showing apoptotic features. *Protoplasma* 216, 31–38.

Decker, E.L., Wiedemann, G., and Reski, R. (2015). Gene targeting for precision glyco-engineering: production of biopharmaceuticals devoid of plant-typical glycosylation in moss bioreactors. *Methods Mol. Biol.* 1321, 213–224.

Goodstein, D.M., Shu, S., Howson, R., Neupane, R., Hayes, R.D., Fazo, J., Mitros, T., Dirks, W., Hellsten, U., Putnam, N., et al. (2012). Phytozome: a comparative platform for green plant genomics. *Nucleic Acids Res.* 40, D1178–D1186.

Gupta R., and Brunak S. (2002). Prediction of glycosylation across the human proteome and the correlation to protein function. *Pac. Symp. Biocomput.* 7, 310–322.

Lang, D., Ullrich, K.K., Murat, F., Fuchs, J., Jenkins, J., Haas, F.B., Piednoel, M., Gundlach, H., Van Bel, M., Meyberg, R., et al. (2018). The *P. patens* chromosome-scale assembly reveals moss genome structure and evolution. *Plant J.* 93, 515–533.

Mark, B.L., Wasney, G.A., Salo, T.J.S., Khan, A.R., Cao, Z., Robbins, P.W., James, M.N.G., and Triggs-Raine, B.L. (1998). Structural and functional characterization of *Streptomyces plicatus β-N*-acetylhexosaminidase by comparative molecular modeling and site-directed mutagenesis. *J. Biol. Chem.* 273, 19618–19624.

Niederau, P.A., Eglé, P., Willig, S., Parsons, J., Hoernstein, S.N.W., Decker, E.L., and Reski, R. (2024). Multifactorial analysis of terminator performance on heterologous gene expression in *Physcomitrella*. *Plant Cell Rep.* 43, 43.

Toepfer R., Schell J., and Steinbiss H.H. (1988). Versatile cloning vectors for transient gene expression and direct gene transfer in plant cells. *Nucleic Acids Res.* 16, 8725.

Weise, A., Rodriguez-Franco, M., Timm, B., Hermann, M., Link, S., Jost, W., and Gorr, G. (2006). Use of *Physcomitrella patens* actin 5′ regions for high transgene expression: importance of 5′ introns. *Appl. Microbiol. Biotechnol.* 70, 337–345.

Zhu, L. (2021). Targeted gene knockouts by protoplast transformation in the moss *Physcomitrella patens*. *Front. Genome Edit.* 3, 719087.

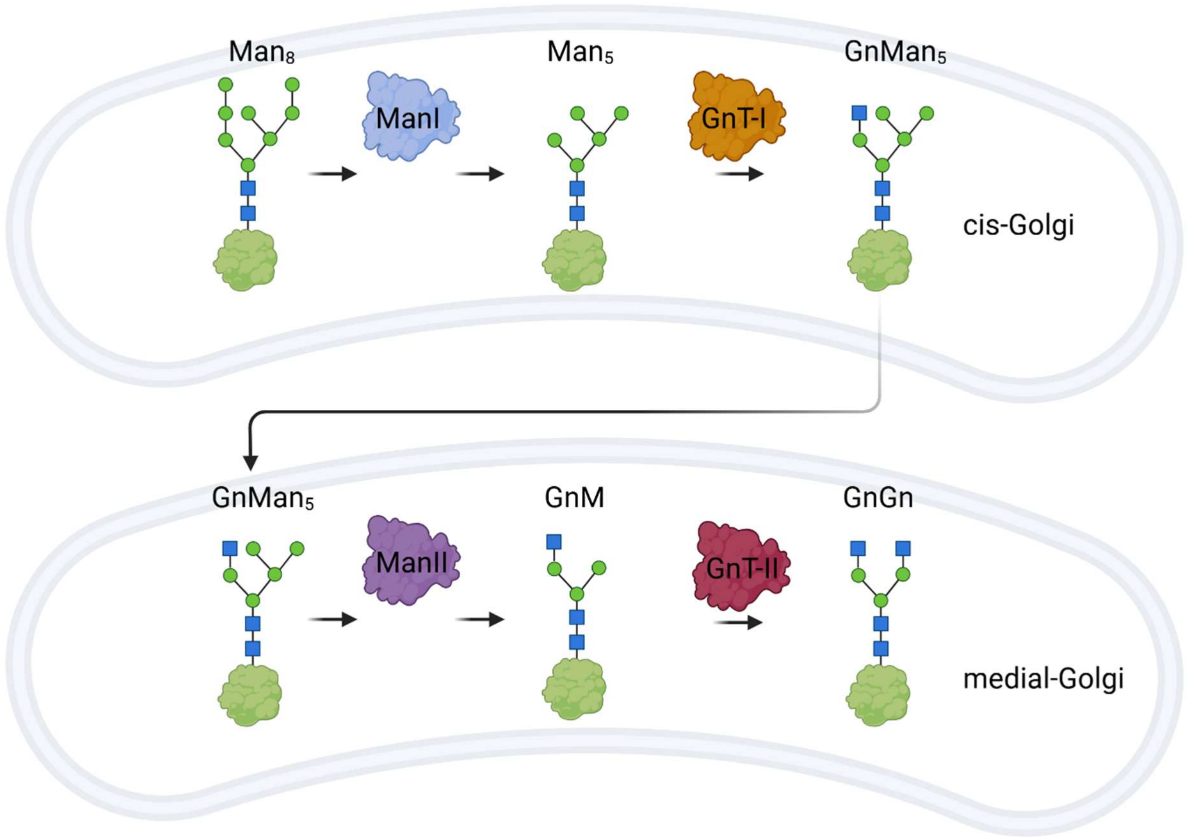

Figure S1: Conserved *N*-glycosylation steps for the conversion of Man_8 glycan structures to GnGn complex-type glycans. *N*-glycans derived from the endoplasmic reticulum contain eight mannose residues (Man_8), the substrate for mannosidase I (ManI) in the *cis*-Golgi. This enzyme trims individual terminal mannose residues in several rounds until the formation of Man_5, which is recognized by *N*-acetylglucosaminyltransferase I (GnT-I). The product of GnT-I is *N*-acetylglucosaminated Man_5 ($GnMan_5$), which is processed further by mannosidase II (ManII) to remove two further terminal mannoses, yielding GnM. *N*-acetylglucosaminyltransferase II (GnT-II) subsequently links another *N*-acetylglucosamine (Gn) to the remaining terminal mannose yielding the GnGn structure. Blue square = *N*-acetylglucosamine and green circle = mannose. Created with BioRender.com/sudh6fn.

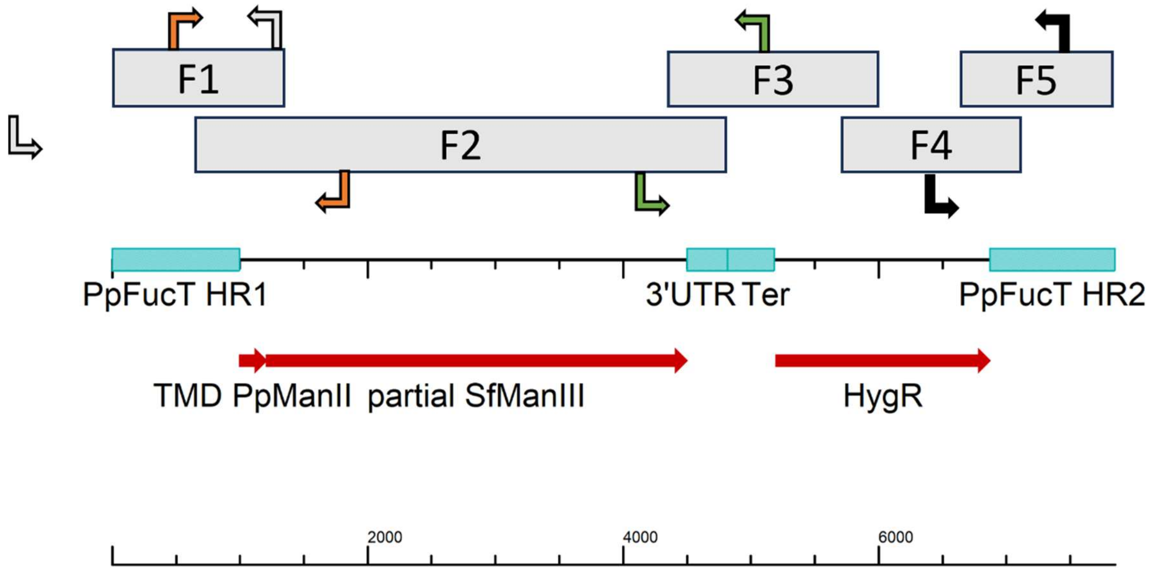

Figure S2: *In vivo* assembly strategy of the expression cassette for partial *Sf*ManIII fused to the transmembrane domain (TMD) of *Pp*ManII for localization in early Golgi vesicles, including the weak *fucT* promoter, terminator (Ter), untranslated region (UTR) and selection cassette conferring hygromycin resistance (HygR). Small PCR fragments (F1–F5) representing different coding regions and genetic elements, including homology regions (HRs) for recombination at the *fucT* locus, were used for the PEG-mediated transfection of moss protoplasts. The HRs covered the upstream and downstream region of the start and stop codons, respectively, to enable the replacement of the entire *fucT* open reading frame with the expression cassette. The sizes of PCR fragments and the distances between primer binding sites (arrows) are not shown to scale. Primers with the same color were used for genotyping by PCR (Table S2).

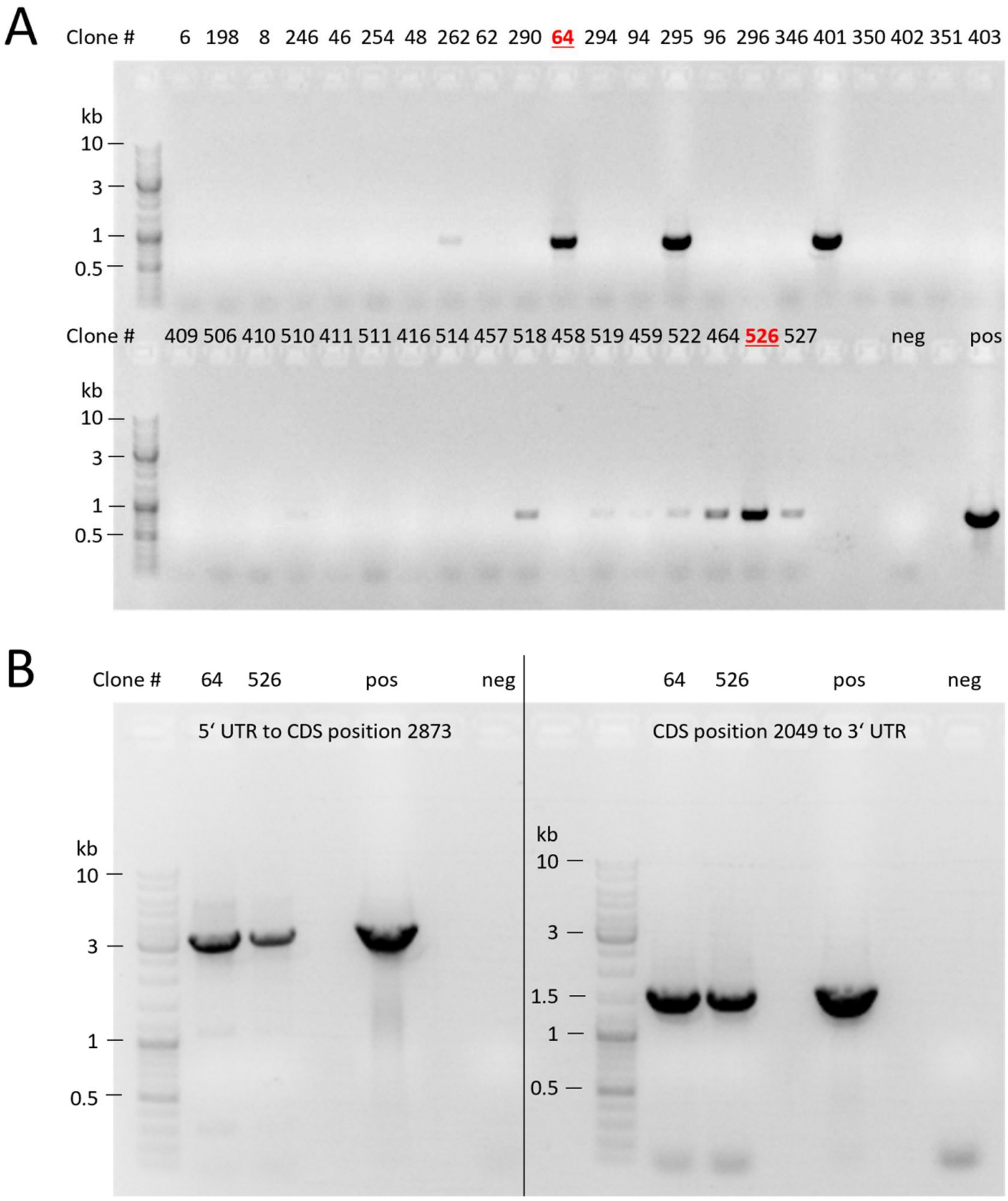

Figure S3: PCR genotyping of clones for the genomic integration of *SfManIII* under the control of a strong promoter. (A) Screening to identify clones with an integrated transgene, including the clones T4#064 (64) and T4#526 (526) highlighted with bold, red and underlined numbers. (B) Confirming integration of the full-length coding sequence (CDS). A fragment was amplified by PCR from genomic DNA spanning the indicated regions of the expression cassette (UTR = untranslated region). Genomic DNA from the parent strain served as the negative control (neg). The transfected DNA shown in Figure 1A was used as the positive control (pos).

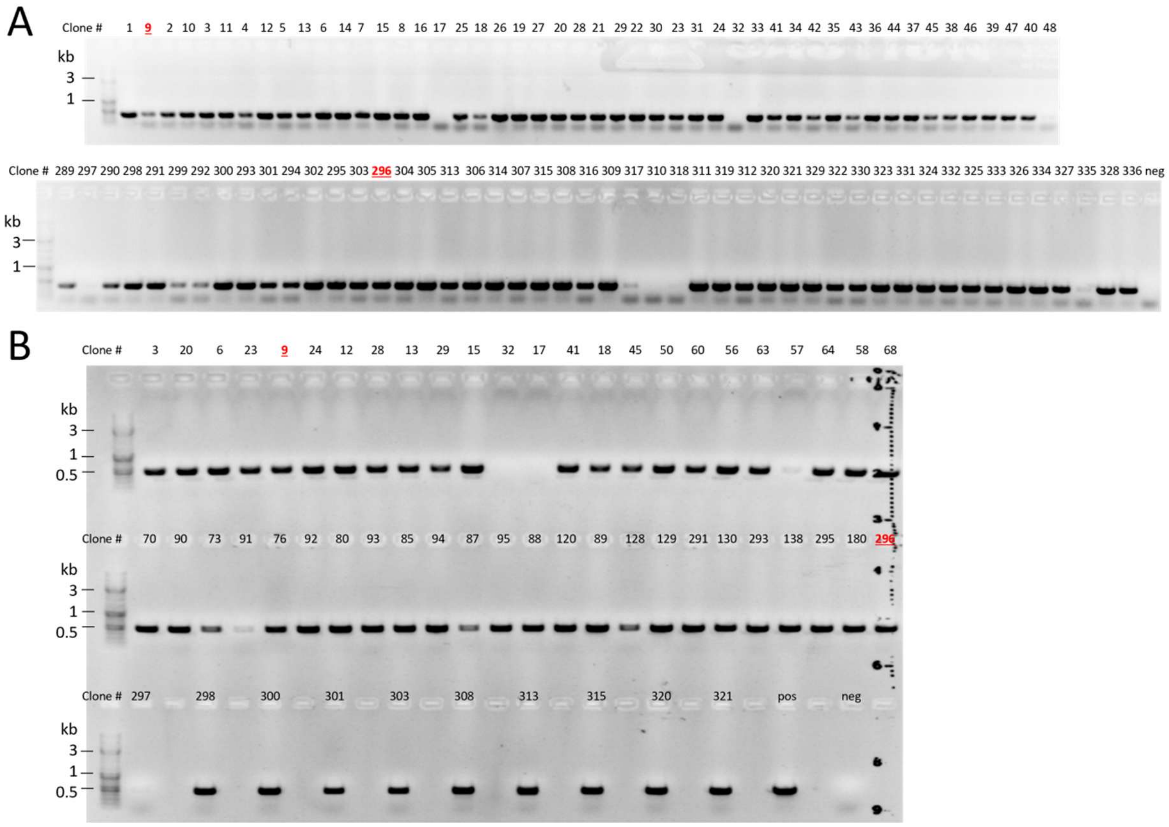

Figure S4: PCR genotyping of clones for the genomic integration of *SfManIII* under the control of a weak promoter. This verifies the assembly of fragments 1 and 2 (A) and fragments 2 and 3 (B) as shown in Figure S2, suggesting full-length integration of the coding sequence. Further characterized clones T9#009 (clone #9) and T9#296 (clone #296) are highlighted with bold, red and underlined numbers. Genomic DNA from the parent strain served as the negative control (neg). The transfected DNA shown in Figure 1A was used as the positive control (pos).

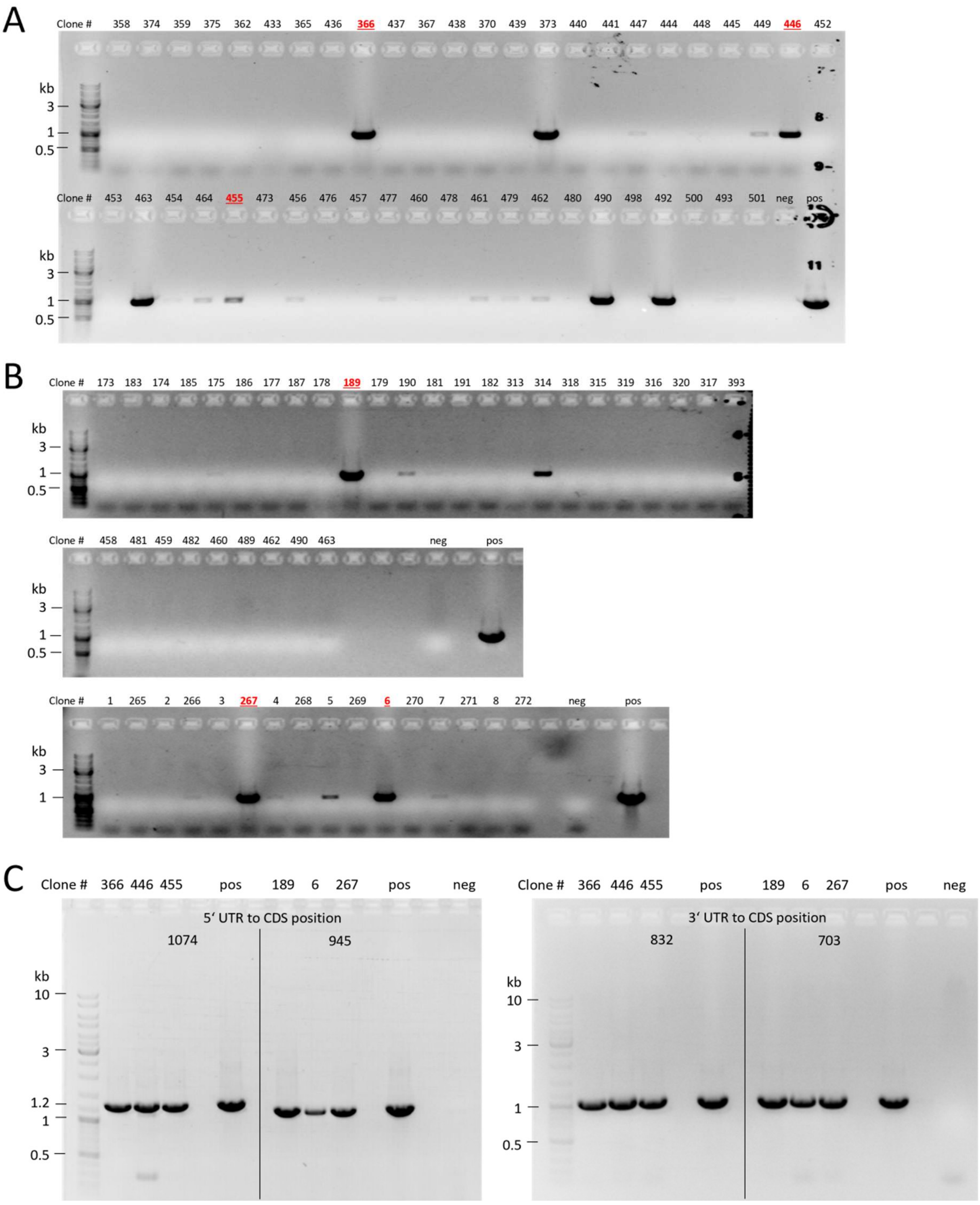

Figure S5: PCR genotyping of clones for the genomic integration of *SfHexo*. Screening to identify clones with an integrated transgene for Golgi localization (A) or secretion (B) of the protein, including clones T1#366 (366), T1#446 (446), T1#455 (455), T3#189 (189), T7#006 (6) and T7#267 (267) highlighted with bold, red and underlined letters. (C) Confirming integration of the full-length coding sequence (CDS). A fragment was amplified by PCR from genomic DNA spanning the indicated regions of the expression cassette (UTR = untranslated region). Genomic DNA from the parent strain served as the negative control (neg). The transfected DNA shown in Figure 1C,D was used as the positive control (pos).

Table S1: Genetic elements used for transgene expression. *P. patens* gene loci refer to genome assembly v3 and annotation v3.3 hosted at Phytozome (Goodstein et al., 2012; Lang et al., 2018). Sequence IDs and brief descriptions are shown above each corresponding sequence.

<table>
<tr><td>Pact</td><td>P. patens actin promoter of Pp3c10_17080V3.3</td></tr>
<tr><td colspan="2">TCCTTGTGACTTTTGTGCATTATTGAGGATATCCATCTATCTAGATTTTGGACAATGTTT
TACTGCCCAAATTTCAATAAGAACCATTCACATATTTGAAACACATTTGATACACTCTA
CATTCATGTCTAGAGTATAGGGACTTGGGTTTAAGATTAGGGTTTCAGATTAGGGCTTGC
AGGGTTACAGTTAAAAGTTAGGATTAAAGATTTAGATGGAGTCTTGGTTCAGAGAGAAAA
AAGGATTTGGGGTAAAGTTTTTATGAAAGAGAATCATCGCCCAAACAAGTAGCGGGACTG
CTGAATGCCTTTTGCAATGAATGAAAATTTATCAACGTCCGTCAATATGTACAAGACCAT
CACATAATGGCCCCCCTGACCACAATTTGAAAAACACACACTTCCTGCCTGGAACCAGTA
ATACAAGTCATTGTAGGGGAGAGAGAGAGAGGGAGAGAGAGCTGTAGCTGCGTATAATAA
GGGTCTCGCAGATTCAGTGCTACGTCGTATGGATACACCGTATCACTTCTGGTGTACAGG
TTACTAAATACTACTCGACACGGGGCGGGCCGATCTGCGGAACGCGCCGGGGCCATGTCC
CAGGGCCCTAGGCCCGCCATATTTCTCTCGTCCACC</td></tr>
<tr><td>5′ UTR</td><td>5′ untranslated region of P. patens actin at Pp3c10_17080V3.3</td></tr>
<tr><td colspan="2">CGGGCCTACGCAAACTTTCCCTTCTCACTTTCCCAGCTCACGCTCTCTGTTCAACGCACA
ACAACGCGTAACCGAGACGGGTTCGGAGCACAAAGTCACCCAGCCCGGCCCGACCCGTGC
CCGTCTGGCGCCTATCTCTCTCCGCCTCTGGGCCCGTTTCGCTCCTGTCCTTGTGTGCTC
TGTCTGGCCCTTCACCGCGCTTCATTGCTTCTTCGACCGAGAGCCTCTTAGCTCCGTCTT
GTTCACCACTGCCGCGGCACTCCGACCCCTTGCATACTCTCTTCTGCGGTGCCTGCTTCT
CCCCATCTCCTGCATCGGTGCCCTGTTGTGTTTTTTTTAAAGGTCAGTCCCTCTATCAC
GTCAGTGTTTCGCATTTCCGTGAAGTGCTCAGGGTTTTTTTGCTGCGAACTGTCGGTGG
AGATGTGCTTTTTGTCGTGTTTGATGTGTGTGCGGTGCAGCGATGGTGGGTTTCTTGGAG
GAGGAGGGAGAGTCTTATTTTAGTCTTGTTGCCCGGTGTGCTCGGGGCGCGAATGTGGGT
TTATGGTAGCGCACAGGTCTGCGTTTGCGATATGTGTGTAGAACCCTGTGCCGAGCGATC
ATCATAATAGTAGTTTCTCGTTTCGGAGGGGCTGGGCTTGTCAAGTGGAACGCAGAGTCG
TAGTTTTGAGAGTTCCAGACGCGCATCGCGCAGCTGTAGTGAGATGTAGCTTCTCGGTGT
GTTTAGTCAAGGTTTCGCTTTTCCGATCTCGGATCATGTTTACGTCCGTCCTTTAAGCTG
GATCTCTTGTTCTTTACAGAACTTGTTCATCGCCCTGACTAAGTTGCTCCAGTGTTGGTC
TGAAGACGACAAGCCTCTTTCTTTCTTGAATAGTAAGAAGAGGAATTTAATCTGAAGGCT
TGTTTTGTACAGTAGTTGGTCGTTTATTCTTTGATGTTTAACTTAGCGTTTCGTTGTACT
TCTACTAATGTACTCTTTAGCTTGGTCCGAGGCTATTATTTAATGAGTCATGCCCTGAAG
TCGGGAACAGCGGGTTGCACCTACAATCATATGGATATGAGGATTCGGGTCGAGTATTAA</td></tr>
</table>

<table>
<tr><td colspan="2">CTTGTAGTCCTTTGTTCATTGTTTTGATTGCGGGGTTTAGCTGGTGCAACTGCCTGAAT
AGCACGCACTGCTTTCCCTGCGTTCGAATCGTCATCAACATTACTATTGTGTAATCCACA
TGGCTACAGCTGCTGTAAGGTTCTGCGTCAAGGGCGTTCTTCAAGAAATAACCTATGTCT
TCCTTGAAATTAAATATTGGTGGTTGTTGTGCAGGTCCGTATTAAATA</td></tr>
<tr><td>SP</td><td>Signal peptide (first 26 amino acids) of the Thuja occidentalis protein at GenBank AY795849.1</td></tr>
<tr><td colspan="2">ATGGCTTTCTATAAGATTTCATCTGTTTTTTCATCTTCTGTTTCTTCTTGATTGCCCTT
CCGTTCCACTCGTACGCC</td></tr>
<tr><td>TMD PpManII</td><td>Transmembrane domain (first 70 amino acids) of P. patens mannosidase II at Pp3c23_10010V3.1</td></tr>
<tr><td colspan="2">ATGGCGGGGGGCTATGGCAGGAGCCTGCCGACGTATTCTTCGTCACTGCCTGGCTCAAAC
CGCAAAGGTTCTAAGCGACGAAGCGCCGGGAAATCTCCGCCAATGTTTAGTTGGAGTTGG
TTTGTGCGCCCTCGGACTTATCTCACGCTCGCGTCGGTAGTGTTCACGTTCCTGTTTCTA
TTCCAAGGTGGGTTCATCGTGACATCGATT</td></tr>
<tr><td>TMD PpFucT</td><td>Transmembrane domain (first 69 amino acids) of P. patens fucosyltransferase at Pp3c11_22030V3.3</td></tr>
<tr><td colspan="2">ATGAAGGGAGATAGGGATACAGGGCGATTTCGCAGGGATGATGCTGCTTTTGAACGGGAT
GTGGAAGGTGGTGAAAGACCCACACCCGGGCTATTAGGCTTGAGGTCTCTGGCATCTAGC
TCAGGACGGGGATGGTGGAGCAAAACTGTTTTGTGGGCCGTATTTGCTGTTGTCCTCATT
GAATGCGCTTTCATTGTTCGCCTTGAT</td></tr>
<tr><td>PpFucT HR1</td><td>Homology region (~1 kb) directly upstream of the start codon of P. patens fucosyltransferase at Pp3c11_22030</td></tr>
<tr><td colspan="2">TTGCTTGTGGATCATTTCGTGATTAACGCGAGCACTGGCTAGGAATACTGGTAAAATTTT
GGCGCGCTAACTCTCTCTATCTCTCTCTGTGTTGCGTTTGATCAGGGGTTTTAGGGTTTG
GGTCCAGGGTTCCGAGGAGTATCGTCACGTGTATTGCGGTCTTGTTGGAGATTCCTCAGT
TGTGCATGTAGATATAAACTTAGTTTAGTCCACGATCGGTTTCTAATCGTGGATTTTTGT
GGGTTTCGGTCGTTGAGCAAGAATTTTGTGAATTTTTTGTATTGGGGGAAGGAAATGGGG
TTATGGCGATATCGTTTTCGTTGGGTTCAACGTGATCGGTGAGCTCCAGGAAGGGCTGGT
CACTCACAATCCGGTATTCGTCTCATCGAGACGCATTTATCGGTTCATTATATGTATATA
TATATATATATATATATATATATGCAGAGTCGATTGTGTTGCAATTTCTGAACTAGGTAC
TGTTGAATTGTAGATTGCCTTCAAGTAGCTCTCGATGTTGGAATGACGGACACAAATTCT
GCTACTGAATGAGACCATATTCTGCACCGTTAATTGGTTTTATGAATATATGGTGTCGAA
TTACATTCTGTCTCGAATCCATGTGCCCTTTCTGCACGAACGTTGGTTTGTAGTTGTAGT
GCAGCCAGTGTGTTTGGTTTAGGATTATGCTTTGACGATCGATGAGTCCGTTTCATGGTT
TTATACTTGTCATTTATCTTCTTGTGATTTTTTGTTTACAAATGTTCCCCCAATTGTAAC</td></tr>
</table>

<table>
<tr><td colspan="2">GTGGGACTTTCGTGTGTGGTGGTTGCTCAAATTGATAGTTTTGGTCATTTGATTTGCGGA
GAGCAATCGGTGTCATGGAAAATCCCTTCGACTGCTTTGATCCAATCAAAGTTCTGCTTG
AGCCAATGTGAGAGGTGGAGGATTGGGCTTCTTCTAAGTGAGGGCTTTCGATTATTGATA
TCTCAAGGCGAATGTTGAAGGCGCTTAGGGAGTAAAT</td></tr>
<tr><td>PpFucT HR2</td><td>Homology region (~1 kb) directly downstream of the stop codon of P. patens fucosyltransferase at Pp3c11_22030</td></tr>
<tr><td colspan="2">TCATATCAGTGTATTATCATCAGTGACTGCATATTGACACCCAATTCTGATGATTTTTA
TTTTTTATTTTTTATTTTTTTTGGTATGGTTACATGCTTTTCAGAGGTTTCTATGCCGCT
GAGTATTTTCCTGAATCGCGAGGTGTGGCAGGTTATCTGCGCCGTCCACCCAATATTTTA
TGATGAGTCGATGATTCGTGAGACTAATCTAGCTTAACCTTTTTCTTACTGGCAAGTCAA
AATTGAGTTTAAAATATTTCAGTATCCTGTTAGTAATTTCAGACACATGTATTCTATGTC
TCATACTCTTTACGTGAAAGTTCAACTGACTTATATTTTGTCGTTTTTCTGTAGATCACT
GTTTTAGCGCATACAAAGACAATTGTCTAAATATTTTTAAAGAAGGTGATATTTTATTAT
AAGATAGAAGTCAATATGTTTTTTTGTTATGCACATGTACTTGAATAAAATAAATTTTTT
TGTTAGATTTAAATACTTTTTGAATTATAGCTTTGTTGAAATTAAGGAATTTATATTCAT
AAGAAGCTACTCGAACAAATTTACAAAGAGAACATTTGATAAGTAAAAGTAATTAAAAGT
TTTTTTTAATTTAAAAAGATTAATTTTTATTAATAAGAAAAATTTGGAAAGTTAGAAAAA
TATTTAACTTTAAAAATTAAGAAAACAAGGCAAAACTTTAATTTACAAATACTTAATGTA
GATTAATTTTCTTATTATATATTAGCACAAATTATCATTATGTGATATTTTATGTTATTG
TTACATATTAAAAAAACACCACACATAGTATAGATAATTTTCTAAAGAAATCATTGCAAT
ATACTTAAAGAAAAGATAAATATCTTTATAATAAGAAAGCTTGGTTATGAGACAAAGAGT
TGAAAAAGTTTTCTCTTACACCTATTGGTTGAGACAAAGAAGTTTTCTTCTTAACCAAAA
ACAGATGTCTTTTTACAAT</td></tr>
<tr><td>SpHexo</td><td>β-N-acetylglucosaminidase S from S. plicatus (GenBank AF063001.3 without start codon)</td></tr>
<tr><td colspan="2">ACCACCGGCGCCGCCCCGGACCGGAAGGCGCCCGTCCGGCCGACGCCCCTCGACCGGGTG
ATCCCGGCCCCCGCCTCGGTCGACCCCGGCGGCGCGCCGTACCGCATCACGCGCGGCACC
CACATCCGCGTCGACGACTCGCGCGAGGCCCGCCGCGTCGGCGACTACCTCGCGGACCTG
CTGCGGCCCGCCACCGGCTACCGGCTCCCCGTCACCGCCCACGGCCACGGCGGCATACGA
CTGCGCCTGGCGGGCGGCCCGTATGGCGACGAGGGCTACCGCCTCGACAGCGGCCCCGCA
GGCGTCACCATCACCGCCCGCAAGGCCGCCGGACTCTTCCACGGCGTCCAGACCCTGCGT
CAGCTGCTGCCCCCCGCCGTCGAGAAGGACTCCGCGCAGCCCGGCCCCTGGCTGGTCGCG
GGCGGCACCATAGAGGACACGCCGCGCTACGCCTGGCGCAGCGCGATGCTCGACGTCTCC
CGGCACTTCTTCGGCGTCGACGAGGTCAAGCGCTACATCGACCGCGTCGCCCGCTACAAG
TACAACAAGCTCCACCTGCACCTCAGCGACGACCAGGGCTGGCGCATCGCCATCGACTCC</td></tr>
</table>

TGGCCGCGCCTGGCGACGTATGGCGGCTCCACCGAGGTCGGCGGCGGCCCCGGCGGCTAC<br>TACACCAAGGCGGAGTACAAGGAGATCGTGCGTTACGCGGCCTCCCGCCACCTTGAGGTG<br>GTCCCCGAGATCGACATGCCGGGCCACACCAACGCCGCCCTCGCCTCCTACGCCGAGCTG<br>AACTGCGACGGTGTGGCGCCCCCGCTGTACACCGGCACCAAGGTCGGCTTCAGCTCGCTG<br>TGCGTGGACAAGGACGTCACCTACGACTTCGTGGACGACGTCATCGGCGAACTCGCCGCG<br>CTCACCCCCGGCCGCTACCTCCACATCGGCGGCGACGAGGCGCACTCCACGCCCAAGGCC<br>GACTTCGTGGCGTTCATGAAGCGGGTGCAGCCGATCGTCGCCAAGTACGGCAAGACGGTC<br>GTCGGCTGGCACCAGCTGGCCGGCGCGGAACCGGTCGAGGGCGCGCTCGTCCAGTACTGG<br>GGCCTGGACCGCACCGGCGACGCCGAGAAGGCCGAGGTCGCCGAGGCCGCCAGGAACGGC<br>ACGGGGCTGATCCTCTCCCCGGCCGACCGGACGTACCTGGACATGAAGTACACCAAGGAC<br>ACCCCGCTGGGCCTGTCCTGGGCGGGCTACGTCGAGGTGCAGCGGTCCTACGACTGGGAT<br>CCGGCCGGCTACCTGCCGGGCGCCCCGGCCGACGCGGTCCGCGGCGTCGAGGCCCCGCTG<br>TGGACGGAGACCCTGTCCGACCCGGACCAGCTGGACTACATGGCCTTCCCGAGGCTGCCG<br>GGCGTCGCCGAACTGGGCTGGTCCCCGGCGTCCACGCACGACTGGGACACCTACAAGGTG<br>CGGCTCGCCGCGCAGGCCCCGTACTGGGAGGCGGCGGGGATCGACTTCTACCGCTCGCCG<br>CAGGTGCCCTGGACCTGA	
**Partial *SfHexo***	**Mature *S. frugiperda* hexosaminidase (minus first 18 amino acids encoding the signal peptide) (GenBank DQ249309.1)**
TTAAGTATCGTTAATCCTGGACCGCAATACCCACCAACGAAGGGGTCAATATGGCCAAGG<br>CCACATCAGCAAACACAAACTGATTCATATTATAAATTAAATCCATCTACTTTTGTAATA<br>ACGGAAAAAGGGAAAACATGCGATATATTAAAAGATGCAATCGATCGGTATATGAAGGTG<br>TTACGAAATACGTACCTGATTGTGGAGAAATATTCAAGAAAACTATCAAGGCATGGAAGT<br>GATGCTGATAATTTTGATGACAACTTTAAGGGAACATTGCAAGAACTACAAATTAACCTG<br>TCAGCACCATGTGAAACTTATCCACACTTGGATATGGATGAGAAATACAGCCTTGATGTT<br>GCCAAGGTATCGGTCCTCAACAGTGACTCCATATGGGGCGTGCTAAGAGGGCTTGAGTCT<br>TTCGTGCAACTGTTTTACATGGCAGACGGATACAAAAATGTGCTTATCAACGCAACACAA<br>ATCCAAGATTTCCCGAAGTACACCCACAGAGGTCTTCTCGTCGATACATCCAGACATTAC<br>ATAACAGTACCAACATTACTGAAAACTTTAGATGCCATGGAAATGAACAAGATGAACGTT<br>TTGCATTGGCACATTGTGGACGACCAGAGCTTCCCTTACAAGAGCGATATGTTCCCTCAA<br>TTGAGTGATGCAGCATACGATCCTACAATGGTATACACCGCTGTTGACATCACCCAAATT<br>GTATCTTATGCAAGACATAGAGGGATACGCGTTCTACCAGAGTTCGATGTGCCAGGCCAT<br>ACCAGCTCCTGGGGCGTTGCGTATCCAAACATCTTAACGAAATGCTACAGTTTAGGACGC<br>GAATTGGGATTGGGACCAATGGACCCAACAAAGAACATCACCTATAAACTGATTGGTGAC<br>TTAATCCGCGAGGTCCAAGAACGGTTCCCAGATAAATATTTTCATGTTGGTGGGGATGAA<br>GTGGAATTGGACTGCTGGATATCAAATTCTGAAATCCGCGATTTCATGAAAGACCACAAC	

ATGACTGATGCTAGTGAACTACACTCATATTTCATGGCTAATGTAATCCCATTGCTGGGA<br>GACAGGTCTAAACCGATTGTGTGGCAAGAAGTATTCGATGAAGGTGTAAGTCTACCCAGC<br>GGCACAATTGTACAAGTATGGAAGAACACGGAAGCTAGAGAAATGCAAAATATCCTAAGT<br>GGCGGTTATAAAGTAATCTATTCATCATCGTGGTATCTGGATCATATTAACGGCGGTGGC<br>GACTGGGCTAAGTATTACGGGGTTGATCCTAGAGAAATAGTCAAAGGAAGTGTCCCTGAA<br>GATAAAGAAGTTGATATTCTGGGAGGTGAAGCATGTATGTGGGGCGAGGTGGTGGATGAC<br>ACAAATATTATTAGCAGGGTATGGCCTCGTGCTAGTGCTGTTGCTGAGGCATTGTGGAGT<br>GGTCACAAATACGAAACAATGCCATATTTAAGGCACTGGTATCAATTCAGAGAAGACTCA<br>GCACACGTAGTAAGCAGCCGTCTTGAAGAACATGCGTGTCGTATGAATCGTCGCGGTATA<br>GAAGCTCAGCCACCAAACGGCCCTGGATTTTGCGTAACAGGGAATTACTTTTAG	
**Partial *SfManIII***	**Partial *S. frugiperda* mannosidase III (minus first 34 amino acids encoding the transmembrane domain) (GenBank AF005034.2)**
TCTCCTCAGAACTATAACAAACCAAGAATCAGTTACCCAGCCAGTATGGAGCACTTCAAA<br>TCTTCCCTCACTCACACCGTCAAGAGCCGAGACGAGCCAACTCCGGATCAATGCCCTGCA<br>TTGAAGGAAAGCGAAGCGGACATCGACACCGTGGCGATATACCCAACTTTTGATTTTCAG<br>CCGAGCTGGTTGCGTACAAAGGAATTTTGGGACAAGTCCTTCGAGGATCGGTATGAAAGG<br>ATTCATAACGACACTACACGGCCTAGACTGAAGGTAATCGTGGTTCCTCACTCACACAAC<br>GACCCGGGATGGCTGAAGACGTTTGAACAGTACTTCGAGTGGAAGACCAAGAACATTATC<br>AACAACATAGTGAACAAACTGCACCAGTACCCCAACATGACCTTCATTTGGACCGAGATA<br>TCGTTTCTGAATGCCTGGTGGGAAAGGTCGCACCCTGTCAAACAAAAGGCATTGAAAAAA<br>CTTATCAAAGAAGGTCGTCTTGAGATCACGACGGGCGGCTGGGTGATGCCGGACGAAGCC<br>TGCACGCATATCTATGCGCTAATTGACCAGTTTATTGAAGGACATCACTGGGTGAAAACT<br>AATCTCGGCGTCATCCCGAAGACAGGATGGTCTATTGACCCCTTCGGCCACGGGGCCACT<br>GTGCCTTACCTGCTAGACCAGAGCGGCCTTGAGGGAACCATTATACAGAGAATCCATTAT<br>GCGTGGAAACAGTGGCTGGCGGAGCGACAGATTGAGGAGTTTTACTGGCTGGCGAGTTGG<br>GCTACTACGAAGCCGTCCATGATAGTGCACAATCAGCCGTTTGATATTTATTCAATAAAA<br>AGCACGTGTGGCCCGCACCCTTCAATTTGTCTCAGTTTCGACTTCAGGAAGATTCCCGGC<br>GAATATTCTGAATACACAGCTAAGCACGAAGACATCACGGAACACAACTTGCACAGCAAG<br>GCAAAGACTTTGATAGAGGAGTACGACCGTATCGGCTCCCTGACTCCACACAACGTGGTG<br>CTGGTGCCGCTCGGAGACGACTTCAGATACGAGTACAGCGTCGAGTTTGATGCCCAATAC<br>GTCAATTATATGAAAATGTTTAACTACATCAATGCTCACAAGGAAATCTTCAACGCTGAC<br>GTACAGTTCGGAACTCCTCTCGATTACTTTAACGCCATGAAAGAAAGACATCAAAATATA<br>CCCAGCTTAAAGGGAGATTTCTTCGTTTACTCCGATATTTTCAGCGAAGGTAAACCAGCG<br>TACTGGTCAGGTTACTACACTACTAGACCCTACCAAAAAATCCTCGCCCGTCAGTTTGAA<br>CACCAACTGCGATCGGCAGAGATTTTATTCACCCTTGTATCGAACTACATCAGACAGATG	

GGTCGCCAAGGAGAGTTCGGAGCTTCTGAGAAAAAGTTAGAAAAATCTTACGAGCAGCTT
ATCTATGCTCGACGGAACTTGGGTCTGTTTCAACATCACGATGCGATTACTGGAACATCA
AAGTCCAGTGTGATGCAAGATTACGGAACCAAACTGTTCACAAGTCTGTATCACTGCATC
CGCCTGCAGGAGGCCGCGCTCACCACCATCATGTTGCCTGACCAGTCGTTGCACTCGCAG
AGCATTATACAAAGCGAGGTTGAGTGGGAAACTTACGGAAAACCGCCCAAGAAGCTGCAA
GTGTCCTTCATTGACAAGAAGAAAGTTATACTTTTTAATCCGTTGGCTGAGACTCGAACT
GAAGTGGTCACGGTTAGATCCAACACGTCCAACATCCGGGTGTACGATACACACAAGAGG
AAGCACGTCTTGTATCAGATAATGCCCAGCATCACAATCCAAGACAACGGCAAGAGTATC
GTAAGCGACACCACGTTCGACATAATGTTCGTGGCCACCATCCCGCCCCTCACCTCCATC
TCGTACAAGCTGCAGGAGCACACCAACACTTCCCACCACTGCGTCATTTTCTGCAACAAC
TGCGAACAATACCAGAAATCCAATGTGTTCCAAATTAAGAAAATGATGCCTGGTGACATA
CAATTAGAAAATGCAGTGCTAAAACTTCTCGTTAATAGGAACACCGGCTTTCTGAGACAA
GTCTATAGAAAGGACATCCGGAAGAGAACTGTCGTTGACGTACAATTCGGCGCATATCAA
AGTGCCCAAAGACATTCTGGTGCTTACCTCTTCATGCCTCATTACGACTCACCTGAGAAG
AATGTTCTGCATCCCTACACTAATCAGAACAACATGCAAGATGATAACATAATCATAGTG
TCCGGACCTATTTCTACGGAAATCACGACCATGTACTTGCCCTTCTTGGTGCACACTATT
AGGATATACAACGTGCCGGACCCGGTACTGTCGCGTGCTATTCTATTAGAGACCGATGTA
GATTTCGAGGCGCCACCTAAGAACAGAGAGACTGAGTTATTTATGAGATTACAGACTGAT
ATACAAAACGGTGACATTCCCGAATTTTACACCGATCAGAACGGATTCCAGTACCAAAAG
AGGGTCAAAGTGAATAAACTAGGAATAGAAGCTAATTACTACCCGATCACTACCATGGCG
TGCCTGCAAGACGAGGAGACCCGGCTCACTCTGCTGACGAACCACGCTCAAGGCGCTGCT
GCATACGAACCAGGACGCTTAGAAGTCATGCTCGATCGTCGAACTCTTTATGATGACTTC
AGAGGAATCGGTGAAGGAGTAGTCGATAACAAACCGACGACTTTCCAGAACTGGATTTTA
ATTGAATCCATGCCAGGCGTGACGCGAGCCAAGAGAGACACTAGTGAACCAGGTTTCAAA
TTTGTTAATGAACGTCGTTTTGGCCCCGGCCAGAAGGAAAGCCCTTACCAAGTACCGTCG
CAGACTGCGGACTACCTGAGCAGGATGTTCAATTACCCGGTGAACGTGTACCTGGTGGAC
ACTAGCGAGGTTGGCGAGATCGAGGTGAAGCCGTACCAGTCGTTCCTGCAGAGCTTCCCG
CCCGGCATCCACCTGGTCACCCTGCGCACCATCACCGACGACGTGCTCGAACTCTTCCCC
AGCAACGAAAGCTACATGGTACTGCACCGACCAGGATACAGCTGCGCTGTCGGAGAGAAG
CCAGTCGCCAAGTCTCCCAAGTTTTCGTCCAAAACCAGGTTCAATGGTCTGAACATTCAG
AACATCACTGCAGTCAGCCTGACCGGCCTGAAGTCACTCCGACCTCTCACAGGTCTGAGT
GACATCCACCTGAACGCTATGGAGGTAAAAACTTACAAGATCAGGTTTTAA

Table S2: Sequences of primers and probes used for PCR genotyping or real-time RT-PCR (qRT-PCR).

**Purpose**	**Sequence 5′→3′**
Genotyping primer 1 for *SfHexo* for clone screening	CAGAGCTTCCCTTACAAGAG
Genotyping primer 2 for *SfHexo* for clone screening	GAAAACGGGACTAAAAGTAATTCCCTGTTACG
Genotyping primer 1 for amplification of 5′ UTR and 5′ half of *SfHexo*	GCAACTGCCTGAATAGCACGCACTG
Genotyping primer 2 for amplification of 5′ UTR and 5′ half of *SfHexo*	TGGGTCCATTGGTCCCAATC
Genotyping primer 1 for amplification of 3′ half of *SfHexo* and 3′ UTR	CAGAGCTTCCCTTACAAGAG
Genotyping primer 2 for amplification of 3′ half of *SfHexo* and 3′ UTR	CGTGCTACGTAATGATAACG
Genotyping primer 1 for *SpHexo* for clone screening	ACGTCTCCCGGCACTTCTTC
Genotyping primer 2 for *SpHexo* for clone screening	GAAAACGGGATCAGGTCCAGGGCACCTG
Genotyping primer 1 for *SfManIII* for clone screening	CCAAGACAACGGCAAGAGTATC
Genotyping primer 2 for *SfManIII* for clone screening	TCTAAGCGTCCTGGTTCGTATG
Genotyping primer 1 for amplification of 5′ UTR and 5′ half of *SfManIII*	GCAACTGCCTGAATAGCACGCACTG
Genotyping primer 2 for amplification of 5′ UTR and 5′ half of *SfManIII*	TCTAAGCGTCCTGGTTCGTATG
Genotyping primer 1 for amplification of 3′ half of *SfManIII* and 3′ UTR	CCAAGACAACGGCAAGAGTATC
Genotyping primer 2 for amplification of 3′ half of *SfManIII* and 3′ UTR	CGTGCTACGTAATGATAACG
Genotyping primer 1 for flanking knock-in region of fragment 1	CATGCGAACGTGGCATAC
Genotyping primer 2 for flanking knock-in region of fragment 1	AGAACCTTTGCGGTTTGAGC
Genotyping primer 1 for fragments 1 and 2 assembled *in vivo*	GTTGTAGTGCAGCCAGTGTG

Genotyping primer 2 for fragments 1 and 2 assembled *in vivo*	AACCAGCTCGGCTGAAAATC
Genotyping primer 1 for fragments 2 and 3 assembled *in vivo*	TTTCGGGTCACGGAACTC
Genotyping primer 2 for fragments 2 and 3 assembled *in vivo*	TACTGCACCGACCAGGATAC
Genotyping primer 1 for fragments 4 and 5 assembled *in vivo*	AAATCACCAGTCTCTCTCTAC
Genotyping primer 2 for fragments 4 and 5 assembled *in vivo*	CAGCGGCATAGAAACCTCTG
qRT-PCR probe for *SfHexo*	TTGCCAAGGTATCGGTCCTCAACAGT
qRT-PCR primer 1 for *SfHexo*	GGATGAGAAATACAGCCTTGA
qRT-PCR primer 2 for *SfHexo*	GGTACTGTTATGTAATGTCTGGA
qRT-PCR probe for *SpHexo*	ACGAGGTCAAGCGCTACATC
qRT-PCR primer 1 for *SpHexo*	GTCTCCCGGCACTTCTTC
qRT-PCR primer 2 for *SpHexo*	AACGCACGATCTCCTTGTAC
qRT-PCR probe for *SfManIII*	TCAAGAGCCGAGACGAGCCAACT
qRT-PCR primer 1 for *SfManIII*	GCACTTCAAATCTTCCCTCA
qRT-PCR primer 2 for *SfManIII*	ACCACGATTACCTTCAGTCTA
qRT-PCR probe for *P. patens actin* as endogenous control	CATGAATCTCACTGCTGCTGCGAACTT
qRT-PCR primer 1 for *P. patens actin* as endogenous control	GCTGGACTGGATGATACTACTG
qRT-PCR primer 2 for *P. patens actin* as endogenous control	TTGGCTGTGCGAGAATCTAA
